# Anatomical characterization of Cre driver mice for neural circuit mapping and manipulation

**DOI:** 10.3389/fncir.2014.00076

**Published:** 2014-07-10

**Authors:** Julie A. Harris, Karla E. Hirokawa, Staci A. Sorensen, Hong Gu, Maya Mills, Lydia L. Ng, Phillip Bohn, Marty Mortrud, Benjamin Ouellette, Jolene Kidney, Kimberly A. Smith, Chinh Dang, Susan Sunkin, Amy Bernard, Seung Wook Oh, Linda Madisen, Hongkui Zeng

**Affiliations:** Allen Institute for Brain ScienceSeattle, WA, USA

**Keywords:** Cre driver mice, genetic tools, anatomical characterization, *in situ* hybridization, neuronal cell types

## Abstract

Significant advances in circuit-level analyses of the brain require tools that allow for labeling, modulation of gene expression, and monitoring and manipulation of cellular activity in specific cell types and/or anatomical regions. Large-scale projects and individual laboratories have produced hundreds of gene-specific promoter-driven Cre mouse lines invaluable for enabling genetic access to subpopulations of cells in the brain. However, the potential utility of each line may not be fully realized without systematic whole brain characterization of transgene expression patterns. We established a high-throughput *in situ* hybridization (ISH), imaging and data processing pipeline to describe whole brain gene expression patterns in Cre driver mice. Currently, anatomical data from over 100 Cre driver lines are publicly available via the Allen Institute's Transgenic Characterization database, which can be used to assist researchers in choosing the appropriate Cre drivers for functional, molecular, or connectional studies of different regions and/or cell types in the brain.

## Introduction

Experimental access to neural components, i.e., the specific cell types and neuronal populations which constitute a circuit, is critical for understanding the complexity of network connectivity and functional roles in perception and behavior. The most advanced set of tools to label, monitor, and manipulate specific cell populations to date are genetically engineered mouse lines and viral vectors, which utilize several common strategies to drive expression of transgenes in cells of interest (Huang and Zeng, 2013). One of the most widely used approaches is the Cre/loxP binary system, in which the Cre recombinase transgene activates a reporter gene through recombination at loxP sites. This genetic strategy is highly flexible, enabling cell type-specific and regional control of Cre expression by unique gene promoters combined with an increasing variety of reporter genes, including fluorescent proteins, optogenetic molecules, and calcium indicators that become active in the presence of Cre (Madisen et al., 2010, 2012; Zariwala et al., 2012; Zeng and Madisen, 2012). Using this binary system, cell-specific transgene expression is accomplished practically through breeding of Cre driver and reporter transgenic mouse lines, co-injection of Cre driver and reporter engineered recombinant viruses into wild type mice, or the injection of recombinant viruses into transgenic mouse lines, which allows for additional spatial and temporal control of reporter gene expression. These approaches are becoming common tools in many neuroscience laboratories and have contributed to significant advances in understanding a variety of specific functional circuits (O'Connor et al., 2013; Ramirez et al., 2013; Anthony et al., 2014).

Large numbers of Cre driver lines with expression in the central nervous system have been generated by projects such as GENSAT (Gong et al., 2007), the NIH Neuroscience Blueprint Cre Driver Network (Taniguchi et al., 2011), at the Allen Institute, and by many individual laboratories. Many of these lines have been made publicly available to the broader neuroscience community through repositories such as Jackson Laboratory and MMRRC. There are two major approaches to genetic engineering of Cre lines, conventional or bacterial artificial chromosome (BAC) transgenics, and genetic targeting (knock-in). Conventional transgenic approaches for Cre expression are generally more straightforward from a technical standpoint, but can result in ectopic expression patterns that do not match expected endogenous gene expression patterns. This is thought to be due to either the lack of all required regulatory elements driving a Cre transgene cassette or random integration into the genome. However, for specifically defined questions, novel expression patterns may prove serendipitous and should not immediately be ruled out as useless. Knock-in techniques use homologous recombination to insert the Cre transgene cassette directly into the endogenous gene locus, sometimes disrupting gene expression depending on the location of the knock-in. Knock-ins generally capture endogenous gene expression patterns better than transgenic strategies, but there are surprising exceptions. Robust interpretation of results using Cre lines generated by either method requires knowledge of whether the Cre-defined cells are in fact the same populations endogenously expressing the corresponding gene. In fact, there may be and often are differences between Cre recombination patterns and the target gene's endogenous expression; it cannot be assumed for any Cre line that the recombined cells are the same cells which normally express the gene from that specific promoter. These differences and similarities can also depend on brain region. The GENSAT project has publicly provided a critical first characterization step for over 250 BAC transgenic lines by showing Cre reporter expression across the brain (http://www.gensat.org). However, there has been limited brain-wide systematic characterization of Cre driver lines and comparison with endogenous gene expression patterns, as many researchers tend to focus on analyzing the expression of Cre in specific brain areas or circuits of interest. Thus, the full potential and utility of the various Cre lines already available for providing genetic access to specific cell populations within networks across the entire brain is likely not completely realized.

Using the pipeline developed first for the Allen Mouse Brain Atlas (ABA) (Lein et al., 2007), we systematically characterized transgenic mRNA expression patterns from 135 Cre driver lines across the entire brain. Here, we present the Transgenic Characterization database and provide an informatics-based and manual analysis of the expression patterns in these Cre lines, summarizing possible ways in which this database can guide researchers selecting Cre lines for research into neural circuits of interest. All image data are publicly available on the Allen Brain Atlas Data Portal (http://connectivity.brain-map.org/transgenic/), and are integrated with other Institute resources.

## Materials and methods

### Transgenic mice

All animal procedures were approved by the Institutional Animal Care and Use Committee at the Allen Institute for Brain Science. Cre lines were generated at the Allen Institute or imported from external sources for characterization. Methods used to generate BAC transgenic and knock-in Cre lines at the Allen Institute have been described previously (Madisen et al., 2010). External sources included Cre lines generated as part of the NIH Neuroscience Blueprint Cre Driver Network (www.credrivermice.org) and the GENSAT project (http://gensat.org/), as well as individual labs. Cre lines were on mixed or various backgrounds, but the majority were crossed to C57Bl6/J mice and maintained as heterozygous lines upon arrival. All Cre driver lines included in this study (*n* = 135) are shown in Supplemental Table 1, along with information on the method of generation (e.g., knock-in or transgenic), availability at public repositories and links to image series data available for each line through the Transgenic Characterization data portal (http://connectivity.brain-map.org/transgenic/). Lines were generated using conventional and BAC transgenic, or knock-in strategies. Knock-ins include either direct insertion of Cre at ATG start site, which disrupts endogenous gene expression, or bicistronic cassettes inserted after the targeted gene, usually in the 3′UTR using IRES, IRES2, or 2A sequences to mediate ribosomal entry or skipping (Bochkov and Palmenberg, 2006; Trichas et al., 2008). The IRES2 sequence (Clontech) is a non-attenuated IRES that could result in higher levels of expression of the downstream gene (e.g., Cre). The 2A sequence used for new lines generated at the Allen Institute (Table 1) was a modified T2A (5′-ggaagcggcgagggcagaggaagtcttctgacatgcggagacgtggaagagaatcccggccctgccccaggctca-3′) or F2A (5′cgggctaagagaggttctggagcaccggtgaaacagactttgaattttgaccttctcaagttggcgggagacgtggagtccaacccagggccc-3′), as indicated in Supplemental Table 1. Lines imported from external sources have been renamed in specific cases to maintain a standard convention across all lines characterized in our pipeline; see Supplemental Table 1. Line names typically follow this order: (1) NCBI symbol for specific gene promoter, (2) an IRES, IRES2 or 2A sequence preceding Cre if present, (3) Cre, and, for all GENSAT lines, the (4) line number given by GENSAT, e.g., Ntsr1-Cre_GN220. Regulatable versions of Cre are noted by modifying “Cre” to “CreERT2” for the tamoxifen-inducible fusion protein (Feil et al., 1997) and “dCre” for a destabilized Cre fusion gene that allows recombination at loxP sites following administration of trimethoprim (Sando et al., 2013).

**Table 1:** **A list of newly generated Cre driver lines at the Allen Institute with a summary overview of the resulting whole brain expression patterns characterized by ISH**.

**New Cre lines**	**Gene name**	**Expression pattern summary**
Adcyap1-2A-Cre	Adenylate cyclase activating polypeptide 1	Cre expression is enriched in restricted populations within the olfactory areas, hippocampus, striatum, thalamus, midbrain, pons, and medulla. Expression is scattered within the isocortex and hypothalamus. Reporter expression is widespread.
Avp-IRES2-Cre	Arginine vasopressin	Expressed in restricted populations within the hypothalamus.
Nxph4-2A-CreERT2	Neurexophilin	Strong expression in locus coeruleus and dorsal medial hypothalamus. Very sparse in other regions of hypothalamus, and layer 6b of cortex and other brain regions.
Pvalb-2A-CreERT2	Parvalbumin	Scattered expression throughout the cortex. Enriched in restricted populations in the cerebellum, medulla, pons, pallidum, and thalamus.
Pvalb-2A-dCre	Parvalbumin	Scattered expression throughout the cortex. Enriched in restricted populations in the cerebellum, medulla, pons, pallidum, and thalamus.
Penk-2A-CreERT2	Preproenkephalin	Weak widespread expression throughout the brain. Strong expression in striatum, olfactory tubercle, and very sparsely in dentate gyrus and cortex.
Rasgrf2-2A-dCre	RAS protein-specific guanine nucleotide-releasing factor 2	Scattered expression throughout the cortex with enriched expression in layers 2/3. Scattered expression in striatum, amygdala, and hypothalamus. Enriched expression in the ventromedial hypothalamic nucleus and the paraventricular nucleus of the thalamus.
Rorb-IRES2-Cre	RAR-related orphan receptor beta	Strong expression in the zonal layer of the superior colliculus and subregions of thalamus. Dense, patchy expression in layer 4 and sparse expression in layer 5 and 6 in cortex. Also expressed in trigeminal nucleus and small patches of cells in cerebellum.
Slc17a7-IRES2-Cre	Solute carrier family 17 (sodium-dependent inorganic phosphate cotransporter), member 7	Strong expression throughout the cortex, olfactory bulb, and anterior olfactory nuclei. Scattered expression in striatum, hippocampus. Enriched in restricted populations in pons, superior colliculus, and anterodorsal nucleus of thalamus.
Snap25-IRES2-Cre	Synaptosomal-associated protein 25	Strong widespread expression throughout the brain.
Sst-Cre	Somatostatin	Sparse, scattered cells throughout most of the brain.
Tac1-IRES2-Cre	Tachykinin 1	Scattered expression in caudate, septum, hypothalamus, midbrain, hindbrain, and cerebellum. Dense expression in accessory olfactory bulb and anterior olfactory nucleus, thalamus, VMH, and midbrain structures such as superior colliculus.
Tac2-IRES2-Cre	Tachykinin 2	Cre expression is enriched in habenula and restricted populations of the hypothalamus. Sparse expression in cortex, hippocampus, and cerebellum.
Trib2-2A-CreERT2	Tribbles homolog 2 (Drosophila)	Enriched in superficial layer 5 neurons of cortex. In scattered cells in the cortex and throughout the brain. Also widespread in vasculature.

### Characterization pipeline for cre mice

**Figure 1:**
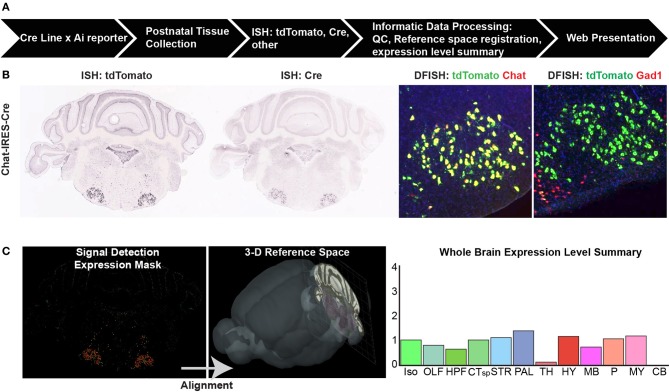
**Cre characterization data types and processing pipeline**. **(A)** An overview of the high-throughput pipeline starting with crosses of mice from every characterized Cre line to a Cre reporter strain (e.g., Ai9, Ai14) to generate double positive mice for brain collection at up to four postnatal ages. The entire brain is sectioned and tissue subjected to *in situ* hybridization (ISH) before informatics data processing and web presentation. **(B)** Types of ISH data collected. For every Cre/reporter line, ISH was used to detect the tdTomato reporter gene at P56. For most Cre lines, additional data types include ISH to detect the Cre gene itself at P56, and double-fluorescent ISH (DFISH) using probes for the tdTomato gene and corresponding endogenous gene to the Cre line promoter or to another cell type marker (e.g., Gad1 for inhibitory neurons). Image series throughout the rostral-caudal extent of the brain were collected to gather whole brain expression patterns. **(C)** Informatics data processing includes signal detection, quantification, and registration of each image series into the 3D Allen Reference Atlas (ARA) space to assign signal (expression energy) to all annotated brain regions within the ARA. A whole brain expression summary is presented online at a coarse level across 12 major brain divisions along with the image series for each brain. Abbreviations are Iso, isocortex; OLF, olfactory areas; HPF, hippocampal formation; CTsp, cortical subplate; STR, striatum; PAL, pallidum; TH, thalamus; HY, hypothalamus; MB, midbrain; P, pons; MY, medulla; and CB, cerebellum.

To systematically characterize whole brain gene expression patterns by *in situ* hybridization (ISH), Cre driver mice from every line were crossed with a reporter strain, typically the tdTomato reporters Ai9 or Ai14 (Madisen et al., 2010). Data analyses in the current study focus on postnatal day 56 (P56) and older mice, but for many of the Cre lines, whole brain characterization was also completed at P4, P14, and P28 and this data can also be found online. We adapted the high-throughput pipeline established for the ABA (Figure 1, Lein et al., 2007), which includes tissue processing, probe hybridization, image capture, and informatics data processing; brief details are described below.

### Tamoxifen and trimethoprim induction

For brain collection at P56, young adult tamoxifen-inducible Cre mice (CreERT2) were treated with ~200 μl of tamoxifen solution (0.2 mg/g body weight) via oral gavage once per day for 5 consecutive days. Trimethoprim (TMP)-inducible Cre mice (dCre) received one i.p. injection of TMP (0.25 mg/g body weight), or one oral gavage dosing of TMP (0.3 mg/g body weight), per day for 1–3 days to activate Cre recombinase. Tissue was collected 1 week after the treatments ended.

### Tissue processing, *in situ* hybridization, and image capture

Mice were deeply anesthetized with 5% isoflurane, and brains rapidly dissected and frozen. Fresh frozen brains were sectioned at 25 μm thickness directly onto slides using a cryostat. Brains were sectioned into eight series in either sagittal or coronal planes, depending in part on expected patterns of expression for a particular Cre line. One series of sections uniformly samples every 200 μm across the brain. One series was used for each probe or probe pair hybridization. Colorimetric ISH (for tdTomato or Cre) and double-fluorescent ISH (DFISH, for tdTomato and other genes of interest) were carried out using previously established and published procedures, with minor differences for DFISH (e.g., TSA biotin was used instead of TSA biotin Plus, and anti-DIG-POD was used at a concentration of 0.10 U/ml instead of 0.25 U/ml) (http://help.brain-map.org/display/mousebrain/Documentation) (Lein et al., 2007; Thompson et al., 2008). Specific information on the probes used to detect the tdTomato and Cre transgenes for ISH and additional probes for DFISH can be found in the Transgenic Characterization database (http://connectivity.brain-map.org/transgenic/). Full slide images of colorimetric ISH were acquired with a 10× objective using the ScanScope (Aperio Technologies, Inc.). DFISH images were captured on VS110 or VS120 systems (Olympus), fully automated, high speed multi-channel fluorescent scanning systems, with a 10× objective.

### Informatics data processing (IDP) pipeline

The IDP has also been described in detail previously (Ng et al., 2007), and was adopted for use in the Transgenic Characterization pipeline. Briefly, each ISH image series undergoes pre-processing (e.g., white-balancing, cropping, and quality control assessment) and then registration to the 3D Allen Reference Atlas (ARA, Dong, 2008) which contains ~800 annotated structures. ISH data are presented as 2D image series on the public Transgenic Characterization database (http://connectivity.brain-map.org/transgenic/), with the ability to “sync” to the ARA, enabling identification of the same approximate positions between different image series and the ARA using the transforms from the 3D registration process. In addition, a signal detection algorithm is applied to segment ISH expression above background for each image and to generate an expression mask (Figure 1C). Each image is divided into 200 μm grids that can be projected back into the 3D ARA space and assigned to specific annotated brain regions. Within each grid, the total intensity of detected pixels is calculated. The expression level per brain structure is measured here as “expression energy,” or the sum of expressing pixel intensity/sum of all pixels for all grids associated with that structure in 3D. A coarse level summary of expression across 12 major brain divisions is presented online for each image series (e.g., Figure 1C, far right).

### Statistical analyses

Statistical analyses were conducted with GraphPad Prism version 6.0. Differences between means were analyzed with two-tailed *t*-tests; *p* < 0.05 was considered significant.

## Results

### Systematic characterization of whole-brain expression patterns from >100 cre driver lines

The Transgenic Characterization pipeline (Figure 1A) was developed to provide data for researchers to evaluate whole brain Cre recombination patterns in a standardized and publicly available format (http://connectivity.brain-map.org/transgenic/). The data consists of high resolution images of ISH for Cre reporters, Cre, and combinations of genes and reporters (e.g., DFISH) on tissue sections covering the entire brain (Figure 1B). Each image series is registered to the 3D ARA, allowing for informatics-based quantification of signal across different annotated brain structures and subsequent data mining (Figure 1C). As of April 2014, we have characterized 135 Cre lines driven by specific gene promoters from various sources (Supplemental Table 1); 37 from GENSAT, 28 from the NIH Blueprint Cre Driver Network (Drs. Z. Josh Huang and Ulrich Mueller), 10 from Dr. Bradford Lowell, 30 from other individual laboratories, and 30 generated at the Allen Institute. Of the Allen Institute lines, here we report for the first time 14 new knock-in Cre drivers (Table 1). The online Transgenic Characterization database also contains data from lines with Cre driven by enhancer elements, a small number of other driver lines (Flp, Dre, tTA, GFP), and reporter lines, which were excluded from the analyses here.

**Figure 2:**
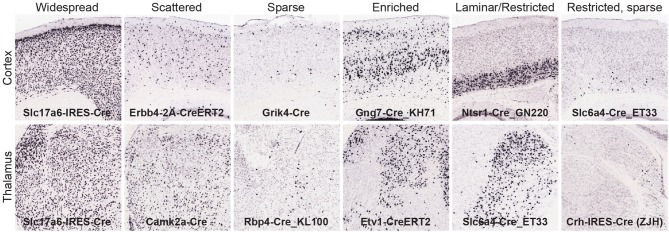
**Manual analysis of Cre reporter expression patterns across the brain**. To aid in interpretation and further mining of the informatics-derived expression energy values, expression patterns were visually classified into one of six categories and recorded in Supplemental Tables 2, 3; (1) widespread, (2) scattered, (3) sparse, (4) enriched, (5) laminar or restricted, and (6) restricted but sparse. Examples from the cortex (top) and thalamus (bottom) are shown for each category.

We derived quantitative values across all gridded voxels in each brain after signal segmentation and registration to the ARA. For this analysis, we focused on calculating the strength of expression in 295 non-overlapping brain structures which tile the entire brain space as defined in the ARA at a mid-ontology level (see Supplemental Table 2, ARA ontology key for structures and abbreviations). Expression strength is related to the number of recombined cells per region. For each of the 135 Cre lines, an exemplar image series of tdTomato reporter expression was chosen; quantitative measurements of reporter expression in each of these 295 brain structures are shown in Supplemental Table 2. These values can be mined to generate a candidate list of brain regions with the highest levels of expression in a particular Cre line. However, due to unavoidable artifacts in segmentation, sometimes poor registration with the ARA, or issues with annotation and border definition within the ARA itself, there can be many false positives on these lists. Therefore, it is critical to also visually inspect the images and areas of interest for Cre expression. We performed a comprehensive visual, manual analysis for a subset of all Cre lines (*n* = 70 lines with complete analyses across all 295 structures, 65 are only partially complete). Each of the 295 structures was identified using the ARA and the “sync” function was used to locate the most closely matched locations in the tdTomato ISH image series. The corresponding tdTomato ISH expression pattern was then classified into one of six patterns (Figure 2); (1) Widespread: very dense expression across neighboring structures (i.e., sharing boundaries) within or across a major brain division, (2) Scattered: less dense expression across neighboring structures, (3) Sparse: low density of expression across neighboring structures, (4) Enriched: some specificity or boundary definition of a particular structure from its neighbors, (5) Restricted (or laminar if in cortex): very specific expression within a particular structure or cortical layer that clearly defines borders with neighboring structures, and (6) Restricted, but sparse: sparse, specific expression within a particular structure. These descriptive categories are included in Supplemental Tables 2, 3, and can be used when mining the quantitative values for visually verified expression and/or determination of enrichment of Cre reporter to specific regions.

### Comparison of whole brain cre expression patterns with endogenous gene expression

**Figure 3:**
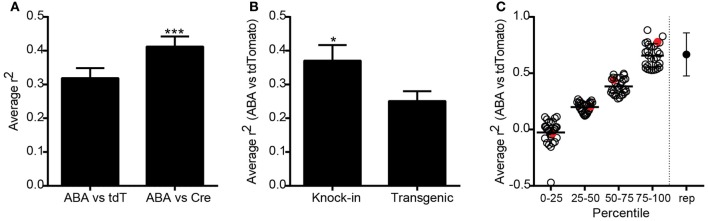
**Overall correlations between Cre expression and corresponding endogenous gene expression across all Cre lines**. **(A)** Expression energy values across 295 annotated structures covering the entire brain were correlated between tdTomato or Cre ISH datasets, and each endogenous gene profile corresponding to the unique promoter driving Cre expression from the Allen Brain Atlas (ABA). The average correlation between ABA and tdTomato datasets was positive (*n* = 119 lines, *r*^2^ = 0.32, Spearman correlation). The average correlation between ABA and Cre ISH datasets was significantly higher (*n* = 83 paired lines, *r*^2^ = 0.41, paired *t*-test ^***^p < 0.001). **(B)** Cre reporter (tdTomato) expression values across the whole brain were significantly more similar to the corresponding ABA gene expression values in knock-in compared with transgenic Cre lines (unpaired *t*-test, *n* = 52 knock-in and 66 transgenic, ^*^p < 0.05). **(C)** Scatter plot showing the ABA vs. tdTomato whole brain *r*^2^-value for each of the 119 Cre lines grouped by percentile rank. The point on the far right shows the average *r*^2^-value between biological replicates within a Cre line for comparison. Red circles indicate examples shown in Figure 4.

To compare expression patterns across the entire brain between genes and the same gene promoter-driven Cre expression, we identified ISH datasets from P56 mice generated as part of the ABA (Lein et al., 2007), which corresponded to the specific promoters of Cre lines in the Transgenic Characterization pipeline. After matching for plane of sectioning (sagittal to sagittal and coronal to coronal), ABA expression data within the same 295 structures were correlated with tdTomato ISH expression values for 119 Cre lines, and with Cre ISH for 83 lines. Each dataset was represented by an *n* = 1–6. Average Spearman rank correlation coefficients were calculated for each line (ABA vs. tdTomato and ABA vs. Cre), and across all Cre lines. The overall correlation was significantly higher when comparing expression of Cre itself to the corresponding ABA expression values, as opposed to the tdTomato reporter (*r*^2^ = 0.41 vs. 0.32, Figure 3A), perhaps due to transient developmental expression of Cre permanently turning on reporter expression. Expression of Cre itself is therefore a better overall indicator of adult Cre expression patterns than the tdTomato reporter, but in general the Cre ISH probe is not as robust at detecting low levels of expression compared to the probe used for tdTomato. Due to this and for the reason that more Cre lines have tdTomato datasets than Cre ISH datasets, subsequent analyses used the tdTomato reporter ISH image series only. Researchers are encouraged to take advantage of the Cre datasets that exist when selecting a particular Cre line. Endogenous gene expression patterns may be more faithfully recapitulated in knock-in mice than those generated using transgenic strategies. We found that the average correlation of tdTomato expression across all Cre lines at P56 with ABA gene expression at P56 was significantly higher in knock-in as compared to transgenic Cre lines (*r*^2^ = 0.37 vs. 0.25, Figure 3B). No significant differences were seen in the average correlation coefficients between this collection of knock-in lines generated using 2A, IRES/IRES2, or direct methods of insertion, or between BAC and conventional transgenics. The average ABA and tdTomato correlation coefficients from each Cre line ranged from −0.47 to 0.88 (Figure 3C). In comparison, the average correlation between biological replicates of tdTomato, Cre, or ABA expression values was 0.57 (shown on far right in Figure 3C). The Cre lines can thus be broken into groups with high (similar to replicate comparisons, above the 75th percentile), mid-level (50–75th percentile), low (25–50th percentile), and very low correlations (below 25th percentile).

**Figure 4:**
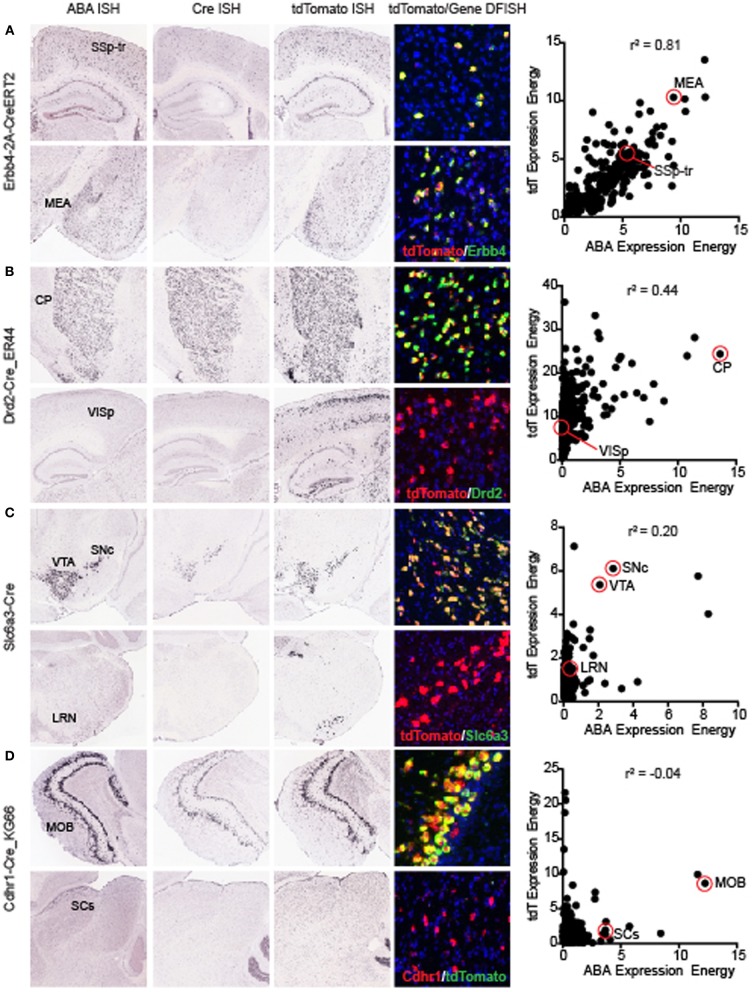
**Representative examples of the correlations between individual Cre lines and the corresponding endogenous gene expression**. Images of ABA, Cre, tdTomato ISH, and tdTomato/endogenous gene DFISH are shown at two positions in the brain (top and bottom panels for each line). For each Cre line, a scatterplot comparing the expression energy in each of 295 brain structures from ABA vs. tdTomato data is shown on the far right. The examples shown here are representative of lines with high **(A)** Erbb4-CreERT2, mid **(B)** Drd2-Cre_ER44, low **(C)** Slc6a3-Cre, and very low **(D)** Cdhr1-Cre_KG66 overall correlations with the corresponding ABA gene expression profiles. In each case shown, DFISH demonstrates that for highly correlated areal gene expression, cell-specific gene expression is also mostly overlapping, whereas when expression energy at the areal level is not correlated, tdTomato and endogenous gene expression are not usually expressed in the same cells. Abbreviations are SSp-tr, primary somatosensory area, trunk region; MEA, medial amygdalar nucleus; CP, caudoputamen; VISp, primary visual area; SNc, substantia nigra, compact part; VTA, ventral tegmental area; LRN, lateral reticular nucleus; MOB, main olfactory bulb; and SCs, superior colliculus, sensory related.

The overall correlation coefficient per Cre line may be impacted by several different factors. Across all Cre lines, recombination patterns relative to the corresponding gene can be roughly classified into three categories; (1) Faithful recapitulation in all brain areas, (2) Faithful recapitulation in areas where endogenous gene is expressed *plus* additional brain areas where it is not, and (3) Faithful recapitulation in a subset of all areas where endogenous gene is expressed. There may also be a mixture of (2) and (3) in some Cre lines. One of the Cre lines with a high correlation coefficient, representing category (1), is the knock-in line Erbb4-2A-CreERT2 (*r*^2^ = 0.81, Figure 4A) (Madisen et al., 2010). Erbb4 is expressed in scattered populations of GABAergic cells in many brain regions including the cortex and hippocampus (Yau et al., 2003). Cre and tdTomato ISH patterns are strikingly similar to ABA when visually inspected. *Erbb4* and tdTomato mRNAs are also highly co-localized within single cells, shown by DFISH. A scatterplot of the expression energy value for each of 295 brain structures in the ABA vs. tdTomato dataset shows the strong positive relationship between the two genes across most structures. Within a Cre line, expression patterns can be similar to the endogenous pattern in most brain structures, but dissimilar in others, e.g., category (2) above. For example, the BAC transgenic Cre line Drd2-Cre_ER44 (Figure 4B) captures the dopamine D2 receptor expression in the caudoputamen, but shows ectopic expression of both Cre and tdTomato in cortical areas. However, it is also possible that the expression of *Drd2* in cortical regions is below the detection level of the *Drd2* ISH. A similar example of Cre reporter expression both within and outside areas where endogenous gene is expressed at P56 is the knock-in Slc6a3-Cre line (Zhuang et al., 2005) (*r*^2^ = 0.2, Figure 4C). Here, strong and highly co-localized expression is observed in the VTA and SNc for both the *Slc6a3* and tdTomato reporter genes, but tdTomato is observed in additional areas, such as the lateral reticular nucleus of the medulla, which does not express detectable *Slc6a3* at any point across development (data not shown, but available at http://developingmouse.brain-map.org/). An example of category (3), where Cre is expressed in a subset of all regions with endogenous gene expression, is seen in the BAC transgenic Cdhr1-Cre_KG66 line (Figure 4D). Cre and the tdTomato reporter are both highly expressed in the olfactory bulb, similar to *Cdhr1*, but not in the sensory region of the superior colliculus (SCs).

### Cre lines allow genetic access to specific brain structures

One main advantage of systematic whole brain expression characterization across many Cre lines is the ability to identify both known and novel Cre lines with expression in specific brain regions and circuits of interest. To enable comparison across Cre lines and mine the tdTomato ISH datasets for Cre lines with expression enriched in any of the 295 brain structures, we calculated the fold change difference between the tdTomato expression value within each structure and the average whole brain signal per Cre line (Supplemental Table 3). Note that this calculation will only highlight those regions that are specifically enriched over the entire brain, and will not highlight areas with expression enriched relative to local subdivisions of the brain, e.g., the lateral geniculate nucleus relative to the thalamus as a whole, although these values could also be generated from Supplemental Table 2. Fold change values can be sorted within each brain structure to identify Cre lines showing the most enrichment to the area(s) of interest. Note also that we did not impose a threshold for expression energy values prior to this fold change calculation, due to the high level of variability between expression energy values that correspond to expressed or not expressed across Cre lines and image series. Therefore, care must be taken in assessing the Cre lines with the highest fold change values as the absolute expression energy is not taken into account, and may in fact be very low or not expressed. The manual, visual inspection notes on expression patterns (described for Supplemental Table 2 and Figure 2) for the set of Cre lines with either full or partial manual annotation can also be used as a preliminary and associated guide to determine whether Cre reporter is expressed or not expressed. The structure columns can be sorted or filtered for “enriched” or “restricted” to identify Cre lines with manually verified anatomically-selective expression patterns. All brain structures have at least one Cre line with detectable expression of Cre reporter, although not all have anatomically-enriched or restricted expression (e.g., they define borders with neighboring nuclei).

**Figure 5:**
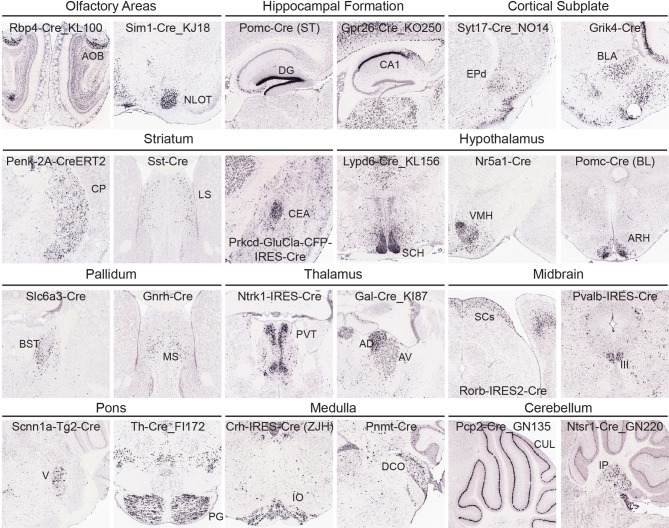
**Cre lines provide genetic access to specific brain areas through enriched or restricted gene expression patterns**. Within this collection of 135 characterized Cre lines, enriched expression patterns can be seen in nuclei from all major brain regions. Representative examples of Cre lines with region-selective expression patterns of the tdTomato reporter are shown within olfactory areas (Rbp4-Cre_KL100, Sim1-Cre_KJ18), hippocampal formation (Pomc-Cre (ST), Gpr26-Cre_KO250), cortical subplate (Syt17-Cre_NO14, Grik4-Cre), striatum (Penk-2A-CreERT2, Sst-Cre, Prkcd-GluCla-CFP-IRES-Cre), hypothalamus [Lypd6-Cre_KL156, Nr5a1-Cre, Pomc-Cre (BL)], pallidum (Slc6a3-Cre, Gnrh-Cre), thalamus (Ntrk1-IRES-Cre, Gal-Cre_KI87), midbrain (Rorb-IRES2-Cre, Pvalb-IRES-Cre), pons (Scnn1a-Tg2-Cre, Th-Cre_FI172), medulla (Crh-IRES-Cre(ZJH), Pnmt-Cre), and cerebellum (Pcp2-Cre_GN135, Ntsr1-Cre_GN220). Abbreviations are: AOB, accessory olfactory bulb; NLOT, nucleus of the lateral olfactory tract; DG, dentate gyrus; field CA1; EPd, endopiriform nucleus, dorsal part; BLA, basolateral amygdalar nucleus; CP, caudoputamen; LS, lateral septal nucleus; CEA, central amygdalar nucleus; SCH, suprachiasmatic nucleus; VMH, ventromedial hypothalamic nucleus; ARH, arcuate nucleus; BST, bed nuclei of the stria terminalis; MS, medial septal nucleus; PVT, paraventricular nucleus of the thalamus; AD, anterodorsal; and AV, anteroventral nucleus of the thalamus; SCs, superior colliculus, sensory related; III, oculomotor nucleus; V, motor nucleus of trigeminal; PG, pontine gray; IO, inferior olivary complex; DCO, dorsal cochlear nucleus; CUL, culmen; and IP, interposed nucleus.

At a coarse level, subcortical regions can be broken down into olfactory areas, hippocampal formation, cortical subplate, striatum, pallidum, thalamus, hypothalamus, midbrain, pons, medulla, and cerebellum. There are between 7 and 43 unique structures within each of these coarse divisions at the 295 structure ARA ontology level described above. Overall, the variety of Cre lines with regionally enriched expression patterns throughout subcortical brain regions is quite rich, providing diverse possibilities for genetic access to specific regions involved in a large number of brain networks. Figure 5 shows examples of enriched or restricted Cre reporter expression patterns in representative structures within each of the major subcortical brain divisions. Note that the vast majority of Cre lines express Cre in multiple regions throughout the brain, but specific areas are highlighted here for each line. For example, in olfactory areas GENSAT BAC transgenic lines Rbp4-Cre_KL100 has expression restricted to the accessory olfactory bulb, while Sim1-Cre_KJ18 shows Cre reporter expression within the nucleus of the lateral olfactory tract. In the hippocampal formation, there are Cre lines with expression restricted to the dentate gyrus [Pomc-Cre (ST), McHugh et al., 2007] and the CA1 region (Gpr26-Cre_KO250). In cortical subplate regions, the dorsal endopiriform nucleus could be accessed using the Syt17-Cre_NO14 line and the basolateral amygdala in the Grik4-Cre line (Nakazawa et al., 2002). Several Cre lines also have region-specific expression within striatal regions, including the caudoputamen. Within the CP, many lines show cell type-selective expression which also defines borders with neighboring areas (e.g., Penk-2A-CreERT2, Drd1-Cre, Drd2-Cre_ER44, Heusner et al., 2008). Specific expression patterns can also be found within the lateral septum (e.g., Sst-Cre) and central amygdala (e.g., Prkcd-GluCla-CFP-IRES-Cre, Haubensak et al., 2010). In the hypothalamus, there are also several examples of Cre lines that have expression patterns which both define an anatomical region's boundaries with other nuclei (e.g., suprachiasmatic nucleus, Lypd6-Cre_KL56) and mark specific cell types (e.g., Nr5a1-Cre in ventromedial hypothalamus, and Pomc-Cre (BL) in the arcuate nucleus, Balthasar et al., 2004; Dhillon et al., 2006). In pallidum regions, Slc6a3-Cre (Zhuang et al., 2005) expresses Cre reporter specifically within the bed nuclei of the stria terminalis and Gnrh-Cre (Yoon et al., 2005) has strong expression within the medial septum. Cre lines are also available which isolate specific thalamic nuclei, including the paraventricular nucleus of the thalamus (e.g., Ntrk1-IRES-Cre) and anterodorsal and anteroventral nuclei (Gal-Cre_KI87). In the midbrain, Rorb-IRES2-Cre directs specific restricted expression to the sensory regions of the SCs and the oculomotor nucleus can be accessed separately from its neighbors using the Pvalb-IRES-Cre line (Hippenmeyer et al., 2005). Nuclei within the pons with restricted expression of Cre include the motor nucleus of the trigeminal (Scnn1a-Tg2-Cre, Madisen et al., 2010) and the pontine gray (Th-Cre_FI172). In the medulla, the inferior olivary nucleus specifically expresses Cre in Crh-IRES-Cre (ZJH) mice (Taniguchi et al., 2011) and the dorsal cochlear nucleus has anatomically restricted expression of Cre in the Pnmt-Cre line (Ebert et al., 2004). Finally, several Cre lines show expression in the cerebellum, including Purkinje cells (e.g., Pcp2-Cre_GN135) in the cerebellar lobules and in the deep cerebellar nuclei (e.g., Ntsr1-Cre_GN220).

### Cre lines for genetic access to specific cell types within cortical circuits

As shown above, a large variety of whole brain expression patterns exist within these 135 Cre lines, but there was an initial selection bias toward including in the database Cre lines with cortical expression, either laminar-restricted Cre or cortical interneurons. Most of these cortical Cre lines also have regionally-specific expression patterns in subcortical brain regions. Each Cre line was placed into a *single* coarse-level group depending on its dominant pattern of expression (Table 2), although many Cre lines actually belong to multiple categories. For example, here we placed several lines with neuropeptide promoters (VIP-Cre, vasoactive intestinal polypeptide, Sst-Cre, somatostatin, and Cck-Cre, cholecystokinin) into the cortical interneuron group as they have been well studied there, but they also belong to the neuropeptide group. Of note, we also characterized very general cell type-specific Cre lines that may be widespread or regionally enriched, including pan-neuronal (e.g., Nes-Cre, Snap25-IRES2-Cre, Eno2-Cre), pan-glial (Gfap-Cre), pan-glutamatergic (e.g., Slc17a6-IRES-Cre, Slc17a7-IRES2-Cre), and pan-GABAergic (e.g., Gad2-IRES-Cre, Slc32a1-IRES-Cre).

**Table 2:** **Cre lines listed only once according to major categorie**.

**Cortical layer selective lines**	**Cortical interneuron lines**	**Neuropeptide/neuromodulator lines**	**Widespread lines**	**Regionally enriched lines**
**A930038C07Rik-Tg1-Cre (layer 5)**	Calb2-CreERT2	Adcyap1-2A-Cre	Dcx-Cre-35	Camk2a-Cre
**A930038C07Rik-Tg4-Cre (layer 5)**	Calb2-IRES-Cre	Agrp-IRES-Cre	Dcx-Cre-38	Camk2a-CreERT2
Chrna2-Cre_OE25 (layer 5)	Cck-CreERT2	Avp-IRES2-Cre	Dcx-CreERT2	Cart-Tg1-Cre
**Chrnb4-Cre_OL57 (layer 2/3/5)**	Cck-IRES-Cre	Chat-IRES-Cre	Eno2-Cre	Cdhr1-Cre_KG66
**Ctgf-Tg1-Cre (layer 6b)**	Cort-T2A-Cre	Chat-IRES-CreER	Gfap-Cre	Cnnm2-Cre_KD18
**Ctgf-Tg2-Cre (layer 6b)**	Crh-IRES-Cre (BL)	Dbh-Cre_KH212	Nefh-Cre	Cyp39a1-Tg1-Cre
**Cux2-Cre (layer 2/3/4)**	Crh-IRES-Cre (ZJH)	Gal-Cre_KI87	Nes-Cre	Cyp39a1-Tg7-Cre
Cux2-CreERT2 (layer 2/3/4)	Dlx1-CreERT2	Gnrh1-Cre	Otof-CreERT2	Dbx1-IRES-Cre
**Cux2-IRES-Cre (layer 2/3/4)**	Dlx5-CreERT2	Hdc-Cre_IM1	Slc17a8-iCre	Drd1a-Cre
Efr3a-Cre_NO108 (layer 5)	Erbb4-2A-CreERT2	Ins2-Cre	Snap25-IRES2-Cre	Drd2-Cre_ER44
Etv1-CreERT2 (layer 5)	Gad2-CreERT2	Kiss1-Cre	Syn1-Cre	Drd3-Cre_KI196
Gng7-Cre_KH71 (layer 2/5)	Gad2-IRES-Cre	Oxt-IRES-Cre	Vamp2-IRES-CreER	Emx1-IRES-Cre
Gpr26-Cre_KO250 (layer 5)	Nkx2-1-CreERT2	Penk-2A-CreERT2		Esr1-2A-Cre
Grp-Cre_KH288 (layer 2/3)	Nos1-CreERT2	Pmch-Cre		Gabra6-IRES-Cre
Htr2a-Cre_KM207 (layer 5)	Pvalb-2A-Cre	Pomc-Cre (BL)		Gabrr3-Cre_KC112
Nr5a1-Cre (layer 4)	Pvalb-2A-CreERT2	Pomc-Cre (ST)		Grik4-Cre
Ntsr1-Cre_GN220 (layer 6)	Pvalb-2A-dCre	Slc6a3-Cre		Grm2-Cre_MR90
Nxph4-2A-CreERT2 (layer 6b)	Pvalb-CreERT2	Slc6a4-Cre_ET33		Kcnc2-Cre
Otof-Cre (layer 6)	Pvalb-IRES-Cre	Slc6a4-CreERT2_EZ13		Lepr-IRES-Cre
Rasgrf2-2A-dCre (layer 2/3)	Slc32a1-IRES-Cre	Slc18a2-Cre_OZ14		Lhx6-CreERT2
Rbp4-Cre_KL100 (layer 5)	Sst-Cre	Th-IRES-CreER		Lypd6-Cre_KL156
Rorb-IRES2-Cre (layer 4)	Sst-CreERT2	Th-Cre_FI172		Mybpc1-Cre
Scnn1a-Tg1-Cre (layer 4)	Sst-IRES-Cre	Ucn3-Cre_KF43		Nefl-IRES-CreER
Scnn1a-Tg2-Cre (layer 4)	Tac1-IRES2-Cre			Ntrk1-IRES-Cre
Scnn1a-Tg3-Cre (layer 4)	Tac2-IRES2-Cre			Oxtr-Cre_ON66
Sim1-Cre_KJ18 (layer 5)	Vip-IRES-Cre			Pcdh9-Cre_NP276
Six3-Cre (layer 4)				Pcp2-Cre (AM)
Syt6-Cre_KI148 (layer 6)				Pcp2-Cre_GN135
Trib2-2A-CreERT2 (layer 5)				Pdzk1ip1-Cre_KD31
Wfs1-Tg2-CreERT2 (layer 2/3)				Plxnd1-Cre_OG1
Wfs1-Tg3-CreERT2 (layer 2/3)				Pnmt-Cre
				Ppp1r17-Cre_NL146
				Prkcd-GluCla-CFP-IRES-Cre
				Satb2-Cre_MO23
				Sim1-Cre
				Slc6a5-Cre_KF109
				Slc17a6-IRES-Cre
				Slc17a7-IRES2-Cre
				Syn1-icre/mRFP1
				Syt17-Cre_NO14
				Tlx3-Cre
				Vipr2-Cre_KE2
				Wnt3a-IRES-Cre

*Lines in bold indicate those for which expression of Cre itself (unlike reporter) shows laminar restriction in the cortex*.

**Figure 6:**
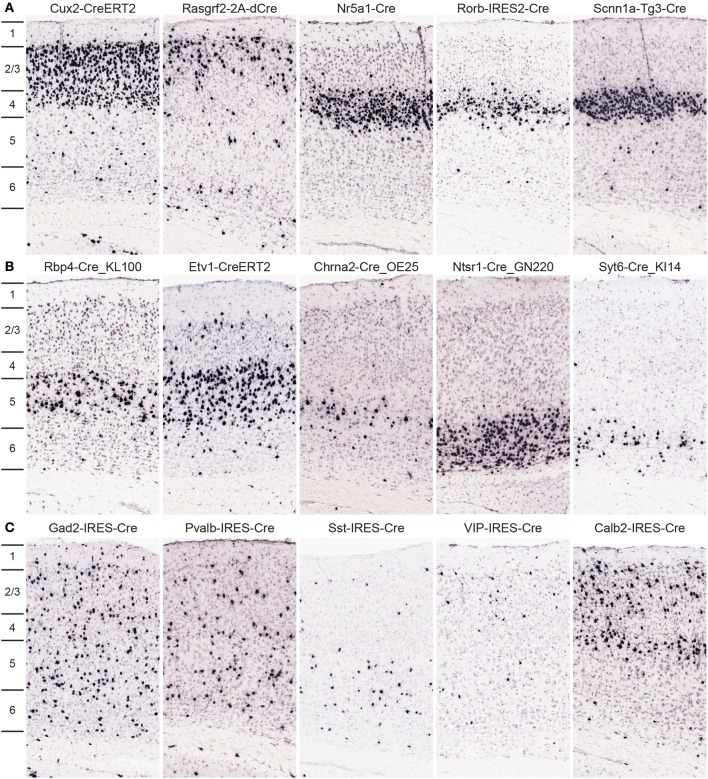
**Cre lines provide genetic access to specific cortical layers and cortical interneurons for circuit analyses**. **(A,B)** Many Cre lines have been generated with selective expression in distinct cortical layers. Representative tdTomato ISH images are shown from a subset of Cre lines with preferential expression in neurons of layers 2/3 (Cux2-CreERT2, Rasgrf2-2A-dCre), layer 4 (Nr5a1-Cre, Rorb-IRES2-Cre, Scnn1a-Tg3-Cre), layer 5 (Rbp4-Cre_KL100, Etv1-CreERT2, Chrna2-Cre_OE25), and layer 6 (Ntsr1-Cre_GN220, Syt6-Cre_KI14). **(C)** Representative images from Cre lines with tdTomato expression driven by cortical interneuron marker promoters (Gad2-IRES-Cre, Pvalb-IRES-Cre, Sst-IRES-Cre, VIP-IRES-Cre, Calb2-IRES-Cre). Density and level of restriction to specific layers varies and is often dependent on the specific cortical region of interest, thus researchers should use the entire whole brain image series to confirm expression in other areas.

Approximately 30 Cre lines were characterized in which expression is highly selective to cells within one cortical layer; 10 example images from the primary somatosensory cortex are shown in Figure 6. Cux2-CreERT2 (Franco et al., 2012) and Rasgrf2-2A-dCre are enriched in cells within superficial layers 2/3/4 or 2/3, respectively. In layer 4, three Cre lines with laminar-selective expression are Nr5a1-Cre (Dhillon et al., 2006), Rorb-IRES2-Cre, and Scnn1a-Tg3-Cre (Madisen et al., 2010). GENSAT lines Rbp4-Cre_KL100 and Chrna2-Cre_OE25, as well as the Etv1-CreERT2 line (Taniguchi et al., 2011) are enriched in layer 5. Layer 6 cells express Cre in the Ntsr1-Cre_GN220 and Syt6-Cre_KI14 lines. For all Cre lines, expression is rarely perfectly restricted to a single layer, but these lines are still incredibly useful for much more precise spatial control of genetic tools for circuit analyses. In addition, most of these Cre lines have roughly equal expression throughout all cortical areas, but a minority are anatomically restricted to specific cortical regions (e.g., Grp-Cre_KH288 is restricted to frontal cortex, Gerfen et al., 2013). Therefore, the whole brain/cortex image series should be viewed by researchers for confirmation in specific regions. Cortical interneuron types within cortical circuits can also be genetically accessed via specific Cre lines, many of which were described previously in the cortex, used successfully for functional studies of circuit components, and for the most part faithfully recapitulate endogenous expression (Taniguchi et al., 2011; Pfeffer et al., 2013; Pi et al., 2013), but are now fully characterized across the brain due to our pipeline processing, including Gad2-IRES-Cre, Pvalb-IRES-Cre, Sst-IRES-Cre, VIP-IRES-Cre, and Calb2-IRES-Cre.

### Cre lines for genetic access to specific neuromodulatory cell types in brain-wide circuits

**Figure 7:**
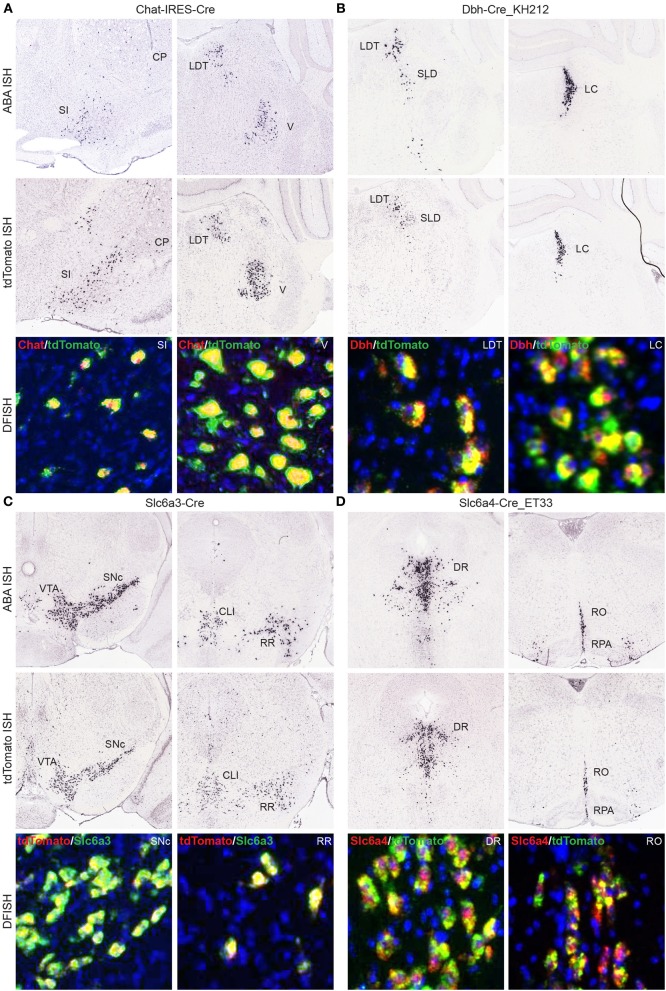
**Cre lines provide genetic access to specific neuromodulatory cell types and brain regions**. Endogenous gene expression and tdTomato reporter ISH images show the faithful recapitulation of regional expression patterns between Cre line reporter expression at P56 and the corresponding endogenous gene expression patterns from the ABA for four major neuromodulatory systems. DFISH data from each line also demonstrates high levels of correspondence between the endogenous gene and reporter expression within the same cell. **(A)** Cholinergic neurons in the SI, basal forebrain; CP, caudoputamen; LDT, laterodorsal tegmental nucleus; and V, motor nucleus of the trigeminal; that express choline acetyltransferase mRNA (*Chat*), among other regions not shown, are also specifically labeled in the Chat-IRES-Cre line. **(B)** Noradrenergic neurons labeled for dopamine beta-hydroxylase mRNA (*Dbh*) are specifically located with the LDT, SLD, sublaterodorsal nucleus; and LC, locus ceruleus. The same regions have enriched expression of the tdTomato reporter in the Dbh-Cre_KH212 transgenic line. **(C)** Dopaminergic cells expressing dopamine transporter mRNA (*Slc6a3*) and Cre reporter mRNA in the Slc6a3-Cre line are found within the VTA, ventral tegmental area; SNc, substantia nigra, pars compacta; CLI, central linear nucleus raphe; and RR, retrorubral area. **(D)** Serotonergic neurons, shown labeled with a probe against the serotonin transporter gene, *Slc6a4*, are located with the dorsal raphe nucleus (DR), and the brainstem raphe nuclei, obscurus (RO) and pallidus (RPA). The same expression pattern is observed in the transgenic Slc6a4-Cre_ET33 line.

Another large group of Cre lines for which we have produced whole brain anatomical expression data include those driven by specific promoters involved in neuromodulatory or neuropeptide signaling. Any Cre line to be used for investigations into cell type-specific roles (as opposed to taking advantage of regional, perhaps serendipitous ectopic expression) should have verified Cre expression patterns that mimic the corresponding endogenous gene expression. In many cases, DFISH data is available in the Transgenic Characterization database to confirm or refute this, or reporter and Cre ISH can be viewed together with ABA data to allow for comparisons of expression within brain regions. Four examples from each of the major neuromodulatory systems where Cre reporter expression matches the corresponding endogenous gene patterns include cholinergic cells expressing Cre in the Chat-IRES-Cre line (Rossi et al., 2011) (Figure 7A), noradrenergic cells expressing Cre in the Dbh-Cre_KH212 line (Figure 7B), dopaminergic cells expressing Cre in the Slc6a3-Cre line (Zhuang et al., 2005) (Figure 7C), and serotonergic cells expressing Cre in the Slc6a4-Cre_ET33 line (Figure 7D).

## Discussion

Promoter-specific Cre lines can provide both cell type and/or regional specificity to label and manipulate network components when used with reporter lines or recombinant viruses. Large scale projects such as GENSAT (Gong et al., 2007; Gerfen et al., 2013) and the NIH Blueprint for Neuroscience Research (Taniguchi et al., 2011) produced a large collection of Cre driver lines in a systematic fashion, but not all lines were comprehensively characterized. GENSAT provided whole brain data by crossing their >250 BAC transgenic Cre lines with a reporter line (Rosa26-EGFP) and presenting images of immunohistological staining on its public website (http://www.gensat.org). We also characterized Cre reporter expression for the collection of lines presented here, but include different and additional data types critical for assessing whole brain recombination patterns in a particular Cre line. Specifically we used ISH to detect reporter and Cre mRNA, and included DFISH for a subset of lines to characterize expression within single cells. We did not include immunohistological analyses of protein levels. Our extensive dataset also benefits from integration with other Allen Institute data, specifically registration with the ARA, links to endogenous gene expression data through the Allen Brain Atlas, and links to related data from other Institute projects. The Transgenic Database presented here is meant as a reference guide for researchers interested in using and identifying Cre driver lines for investigation into specific cell populations and networks.

Two simple, yet powerful, searches made possible through our database and the tables provided here are: (1) Where in the brain is Cre expressed in my line of interest; and (2) Are there driver lines with Cre expression in my area(s) of interest? For the first question, users can view 2D image series (e.g., tdTomato reporter, Cre, or DFISH) sampled across the entire brain for each Cre driver line side-by-side with the ARA to help identify brain structures. In addition, we provide here (Supplemental Table 2) expression values from 295 brain areas per Cre line, which can be sorted to identify regions with the highest Cre expression per line, and also supply associated visual descriptions of these expression patterns for a subset of all lines. Supplemental Table 3 makes it possible to perform the second search by providing fold change ratios of expression value per structure normalized to the average whole brain expression value. High fold change ratios in a particular structure indicate enrichment in that structure over the rest of brain. Again, we provide visually annotated categories to describe expression patterns (and note when there is no expression) associated with the informatics values, but users should also refer to the images online to confirm for themselves.

Major considerations when using Cre driver lines are common across various experiments investigating the roles of cell populations and circuit function, and several of them can be addressed using the Transgenic Characterization resource. One is whether Cre expression faithfully represents endogenous gene expression patterns. We present two data types to aid in this determination: (1) comparison of regional expression patterns between the Cre line and the corresponding gene in the ABA; or (2) DFISH to assess co-localization of Cre reporter and the corresponding endogenous gene mRNAs within the same cells. We performed correlation analyses using the informatics-derived expression values assigned from each data type (ABA or Transgenic Data). There was a wide range of results, with some Cre lines very faithfully capturing whole brain expression patterns, and others performing poorly or at intermediate levels. Lines with lower overall correlation coefficients generally captured a combination of all areas with endogenous gene expression plus ectopic regions, or captured a subset of all endogenous regions. Knock-in lines had expression patterns better predicted from the endogenous gene expression, but still showed variation in results.

A second consideration that can be addressed using this resource is whether reporter expression is consistent with Cre expression (or endogenous gene expression) at the desired age for experiments. When viewing reporter expression at P56, it is important to bear in mind that transient developmental expression of Cre can contribute to seemingly “ectopic” patterns of expression. Indeed, we found that the overall correlation between Cre itself and ABA across all lines was significantly higher than with tdTomato reporter ISH. Cre ISH data in P56 mice is supplied for ~60% of the 135 driver lines characterized here, and should be consulted when available to confirm adult expression of Cre itself. However, transient developmental expression does not account for all seemingly ectopic expression patterns (e.g., see Figure 4B, Cre and tdTomato ISH in cortex, but not in ABA). Care must also be taken in interpreting negative ISH results; very low levels of mRNA may not be detectable by this method. Ectopic expression may also provide serendipitous access to specific circuits. For example, the Ntsr1-Cre_ GN220 line has strong Cre and reporter expression in layer 6 of the cortex, but *Ntsr1* is not endogenously expressed in those cells (at least as detectable by ISH), even so the line has already been used to uncover a role for layer six neurons in gain control in visual cortex (Olsen et al., 2012).

More research and data types will be required to realize the full potential and limitations of available Cre driver lines for genetic access to specific cell types and regions. For example, characterization of the axonal projections originating from different Cre lines provides additional information on whether a subtype of projection neuron is labeled (Gerfen et al., 2013). We are actively pursuing this method of characterization now in the context of mapping whole brain projections using Cre drivers, by injecting Cre-dependent rAAV tracer into brain regions with enriched or restricted expression in over 100 Cre lines as part of our Allen Mouse Brain Connectivity Atlas project (http://connectivity.brain-map.org) (Oh et al., 2014).

Cre lines may be among the best available tools for systematically generating a census of cell types in the mouse brain by providing access to genetically defined cell populations, although it cannot be assumed that a Cre driver equals a single cell type. Many characteristics, or combinations of characteristics, can be used to classify cells into types, including gene expression, morphology, physiological properties, and connectivity. Hetero- or homo-geneity of cell type characteristics within a particular Cre line can be determined through direct labeling using Cre reporters in combination with various experimental techniques; e.g., (1) morphological reconstruction of Cre+ cells (Rotolo et al., 2008), (2) FACS sorting or single cell collection of labeled Cre+ cells for genetic profiling (Siegert et al., 2012; Pfeffer et al., 2013), (3) electrophysiological recording of fluorescent Cre+ cells *in vitro* or *in vivo* (Miyamichi et al., 2013; Runyan and Sur, 2013), and (4) connectivity profiling in local circuits by dual patching of Cre+ and/or Cre- cells (Pfeffer et al., 2013). Analyses at the single cell level will inform us as to whether and which Cre lines label heterogeneous cell populations, as most cell types are not likely defined by single genes. Already genetic strategies are in use to capture more homogenous cell populations using Cre/Flp intersectional techniques (Dymecki et al., 2010); the generation of more Flp drivers will aid this process. Knowledge of specific developmental lineages and timing of Cre induction can also be used to isolate some pure cell types (Taniguchi et al., 2013).

Regardless of whether a Cre line provides genetic access to a “single” cell type, they are commonly used together with a variety of reporter genes for analyses of gene function and the role of different components within specific neural circuits (Gore and Zweifel, 2013). Using Cre lines to target specific cell populations has greatly enhanced our knowledge of the dynamic organization of neural circuits, obtained from tools to monitor and manipulate neural activity (Atasoy et al., 2008; Madisen et al., 2012; Zariwala et al., 2012). Full characterization of Cre expression and understanding the variability of different characteristics in specific gene promoter driver lines will continue to improve the analytical potential of various tools in dissecting circuit functions, and aid in the classification of cell types.

## Author contributions

Julie A. Harris, Seung Wook Oh, Susan Sunkin, Staci A. Sorensen, Amy Bernard, Linda Madisen, and Hongkui Zeng contributed to overall project design and management. Karla E. Hirokawa, Julie A. Harris, Staci A. Sorensen, Phillip Bohn, Marty Mortrud, and Benjamin Ouellette, were main contributors to data and image analysis. Linda Madisen, Maya Mills, Hong Gu, and Hongkui Zeng were responsible for generation of new Cre lines. Lydia L. Ng and Chinh Dang conducted informatics data processing and web presentation. Amy Bernard, Kimberly A. Smith, and Jolene Kidney performed managerial roles in ISH, imaging, and mouse colony planning processes. Julie A. Harris wrote the manuscript with input from other authors.

### Conflict of interest statement

The authors declare that the research was conducted in the absence of any commercial or financial relationships that could be construed as a potential conflict of interest.
